# Structurally simplified biphenyl combretastatin A4 derivatives retain *in vitro* anti-cancer activity dependent on mitotic arrest

**DOI:** 10.1371/journal.pone.0171806

**Published:** 2017-03-02

**Authors:** Daniel Tarade, Dennis Ma, Christopher Pignanelli, Fadi Mansour, Daniel Simard, Sean van den Berg, James Gauld, James McNulty, Siyaram Pandey

**Affiliations:** 1 Department of Chemistry and Biochemistry, University of Windsor, Windsor, Ontario, Canada; 2 Department of Chemistry and Chemical Biology, McMaster University, Hamilton, Ontario, Canada; University of Sheffield, UNITED KINGDOM

## Abstract

The *cis*-stilbene, combretastatin A4 (CA4), is a potent microtubule targeting and vascular damaging agent. Despite promising results at the pre-clinical level and extensive clinical evaluation, CA4 has yet to be approved for therapeutic use. One impediment to the development of CA4 is an inherent conformational instability about the ethylene linker, which joins two aromatic rings. We have previously published preliminary data regarding structurally simplified biphenyl derivatives of CA4, lacking an ethylene linker, which retain anti-proliferative and pro-apoptotic activity, albeit at higher doses. Our current study provides a more comprehensive evaluation regarding the anti-proliferative and pro-apoptotic properties of biphenyl CA4 derivatives in both 2D and 3D cancerous and non-cancerous cell models. Computational analysis has revealed that cytotoxicity of CA4 and biphenyl analogues correlates with predicted tubulin affinity. Additional mechanistic evaluation of the biphenyl derivatives found that their anti-cancer activity is dependent on prolonged mitotic arrest, in a similar manner to CA4. Lastly, we have shown that cancer cells deficient in the extrinsic pathway of apoptosis experience delayed cell death following treatment with CA4 or analogues. Biphenyl derivatives of CA4 represent structurally simplified analogues of CA4, which retain a similar mechanism of action. The biphenyl analogues warrant *in vivo* examination to evaluate their potential as vascular damaging agents.

## Introduction

**Fig 1:**
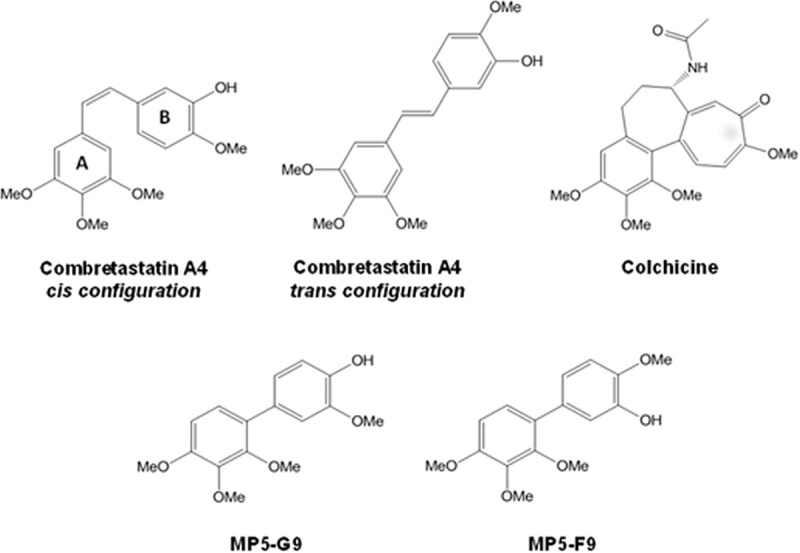
Structure of CA4 and related anti cancer coumpounds.

Combretastatin A4 (CA4; Fig 1) is a *cis*-stilbene that was first isolated from the South African bushwillow tree in 1989 [1]. CA4 and its related compound, colchicine (Fig 1), belong to a class of compounds referred to as colchicinoids. In the past several decades, CA4 has been found to be a potent microtubule targeting agent (MTA) and capable of drastically inhibiting cancer cell proliferation *in vitro*. Excitingly, *in vivo* studies have revealed that CA4 is a vascular disrupting agent (VDA), which is a classification that refers to compounds that can destroy newly formed vasculature, such as found in tumour environments [2, 3]. Clinical studies with a more soluble phosphate derivative of CA4 (CA4P) have revealed an ability to regress tumor vasculature in a variety of cancers. Furthermore, CA4P is well-tolerated, lacking side effects that are common to MTAs, such as immunosuppression [4, 5]. Importantly, the mechanism of CA4’s selectivity is not fully known, as related MTAs, such as colchicine, are only able to exhibit vascular damaging effects at doses that are near its maximum tolerated dose [6]. However, CA4P has been found to be a poor monotherapy, with few instances of objective response in patients, but studies are ongoing and CA4P was recently granted fast track status by the FDA for treatment of platinum-resistant ovarian cancer [7–9]. Additionally, clinical research has begun examining the effectiveness of CA4P as an adjuvant therapy [10].

It has been hypothesized that CA4P’s failure to induce tumor regression in human patients stems from its short half-life in the human body, typically only two to three hours, and its inherent conformational instability [11, 12]. CA4 possesses maximal activity when the two aromatic rings are in *cis* orientation (Fig 1). Importantly, it has been found that CA4 can spontaneously isomerize to the virtually inactive *trans*-stilbene (Fig 1) under the influence of light, heat, or protic media [6]. Thus, medicinal chemists have focused on synthesizing analogues of CA4 with improved pharmokinetic profile and/or conformational rigidity. To date, hundreds of analogues have been synthesized. A common stratagem for combating the conformational instability has been the incorporation of the ethylene linker into a ring system, which prevents spontaneous isomerization [13–16]. The use of ring systems for the synthesis of *cis*-restricted isomers of CA4 has been comprehensively reviewed [17].

We have recently synthesized two biphenyl analogues of CA4 (Fig 1), which feature directly connected aromatic rings [18]. Although our preliminary work suggests that they require doses that are roughly 1000 times higher than CA4 for activity, they represent the first instances of anti-cancer CA4 analogues comprised of directly connected phenyl rings [18]. The structural simplicity prevents inactivating isomerization and the biphenyl derivatives can serve as scaffolds for the further optimization of the CA4 pharmacophore in the search for novel VDAs. In this study, we aim to comprehensively evaluate the activity of biphenyl analogues of CA4 in various *in vitro* models as a proof-of-concept that structurally simplified analogues of CA4 can retain anti-proliferative and pro-apoptotic properties. However, future *in vivo* studies are required to evaluate the potential vascular damaging properties of biphenyl CA4 analogues.

Although much of CA4 research has focused on the synthesis of novel analogues, biochemical inquiry into the precise mechanism of CA4-mediated cell death *in vitro* and *in vivo* has also been conducted. The phenotypic response of cancer cells to CA4 treatment *in vitro* has been disruption of the microtubule network, prolonged mitotic arrest, mitochondrial depolarisation, release and activation of pro-apoptotic proteins, and, ultimately, cell death via apoptosis [19–21].

To date, CA4 has not been shown to have any additional direct cellular targets other than tubulin. Despite the absence of additional cellular targets, inhibition studies have failed to implicate a single cell death pathway. Although apoptosis inducing factor (AIF) has been shown to be released from mitochondria following CA4 treatment, inhibition of poly (ADP-ribose) polymerase **(**PARP), which is necessary for AIF nuclear translocation, fails to rescue cancer cells from dying [20]. Similarly, caspase nine and three have been shown to be cleaved and activated by CA4 treatment, but various caspase inhibitors have failed to prevent cell death [19, 20]. Thus, a single pathway has not been implicated in apoptosis induced by CA4, suggesting redundancy and other relevant cell death pathways. This hypothesis is supported by the observation that multiple analogues of CA4 have been found to retain anti-cancer activity that is seemingly independent of microtubule inhibitory activity [22–26]. Several such CA4 analogues have been compiled in a review [27]. Thus, it remains a distinct possibility that CA4 itself can induce apoptosis in cancer cells independently of microtubule targeting. To test the importance of mitotic arrest in CA4-mediated cell death, which is related to CA4’s ability to disrupt microtubule dynamics, a chronology of cellular events following CA4 treatment was examined. Additionally, small-molecule inhibition of mitotic arrest was utilized to further probe the necessity of mitotic arrest during CA4-mediated cell death. To explore the role of non-tubulin targeting during *in vivo* treatment of tumours with CA4, further studies would need to be conducted.

## Experimental procedures

### Reagents

Two biphenyl analogues (MP5-F9 and MP5-G9) of CA4 were prepared as previously reported [18] and their biological activity was compared to CA4 (Sigma-Aldrich Canada, Cat. No. C7744, Mississauga, ON, Canada). Reversine (Sigma-Aldrich Canada, Cat. No. R3904, Mississauga, ON, Canada) and RO-3306 (Sigma-Aldrich Canada Cat. No. SML0569, Mississauga, ON, Canada), small molecule inhibitors known to prevent mitotic arrest, were used to test the causal connection between mitotic arrest and apoptosis. These compounds were dissolved in dimethyl sulfoxide solvent (DMSO) at stock concentrations of either 10 μM, 1 mM, or 10 mM and were further diluted in phosphate buffer saline (PBS) before treatment of cells.

### Cell culture

E6-1 jurkat cells (ATCC, Cat. No. TIB-152, Manassas, VA, USA), an acute T-cell leukemia cell line, as well as a jurkat cell line dominant negative for the Fas-Associated Death Domain (FADD) protein (DN-FADD Jurkat; ATCC, Cat. No. CRL-2572, Manassas, VA, USA), were cultured with RPMI-1640 (Sigma-Aldrich Canada, Mississauga, ON, Canada) supplemented with 10% (v/v) FBS standard (Thermo Scientific, Waltham, MA, USA).

Chronic myelomonocitic leukemia cancer cells, MV-4-11, were obtained from American Type Culture Collection (Cat. No. CRL-9591, Manassas, VA, USA). MV-4-11 cells were cultured with Iscove's Modified Dulbecco's Medium (ATCC, Cat. No. 30–2005, Manassas, VA, USA) supplemented with 10% (v/v) FBS standard.

Peripheral blood mononuclear cells (PBMCs) were also tested. Briefly, whole blood was collected from healthy volunteers in BD Vacutainer^®^CPT^TM^ Tubes with Sodium Heparin^N^ (Becton, Dickinson and Company, Cat. No. 362753, Franklin Lakes, NJ, USA) at room temperature. Immediately, the collection tubes were inverted 5 times and centrifuged (30 minutes, room temperature, 1500–1800 x g). The layer of PBMCs under the plasma layer in each tube was collected, pooled together, resuspended in 50 mL of PBS, and centrifuged (15 minutes, room temperature, 300 x g). The supernatant was aspirated without disrupting the pellet prior to resuspending PBMCs in RPMI-1640 medium supplemented with 10% (v/v) FBS standard.

The MG-63 osteocarcoma cell line (ATCC, Cat. No. CRL-1427, Manassas, VA, USA) were cultured with Eagle’s Minimum Essential Medium (Sigma-Aldrich Canada, Mississauga, ON, Canada) supplemented with 10% (v/v) FBS standard.

Saos-2, an osteocarcoma cell line (ATCC, Cat. No. HTB-85, Manassas, VA, USA), was cultured with McCoy’s 5A Medium Modified (Sigma-Aldrich Canada, Mississauga, ON, Canada) supplemented with 15% (v/v) fetal bovine serum (FBS) standard.

Two human colorectal cancer cell lines, HT-29 (ATCC, Cat. No. CCL-218, Manassas, VA, USA) and HCT-116 (ATCC, Cat. No. CCL-247, Manassas, VA, USA), were cultured with McCoy’s 5A Medium supplemented 10% (v/v) FBS standard.

Non-cancerous cell lines were also utilized to evaluate the selectivity of the cancer selectivity of CA4 and novel CA4 analogues. Normal-derived colon mucosa cells (NCM460) were obtained from INCELL, San Antonio, Texas [28]and were grown in RPMI-1640 medium supplemented with 10% (v/v) FBS standard.

Non-cancerous human osteoblasts (Hob; Cell Applications, Inc., Cat. No. 406-05a, San Diego, CA, USA) were cultured in Osteoblast Growth Medium (Cell Applications, Inc., Cat. No. 417–500, San Diego, CA, USA).

All media was further supplemented with 10 mg/mL gentamicin (Gibco BRL, VWR, Mississauga, ON, Canada). Cells were cultured at 37°C and at 5% CO_2_.

### Quantification of cellular proliferation

To evaluate the cellular proliferation as a function of active cellular metabolism, a WST-1 assay was performed according to the manufacturer's protocol (Roche Applied Science, Cat. No. 11644807001, Indianapolis, IN, USA). Cells were plated in 96-well clear bottom tissue culture plates and incubated for 24 hours. Seeding density was 6 x 10^3^ cells/well for Saos-2, 1.8 x 10^4^ cells/well for MV-4-11, 1 x 10^4^ cells/well for E6-1, 2.5 x 10^3^ cells/well for MG-63, 4 x 10^3^ cells/well for HOb, 2 x 10^3^ cells/well for HT-29, 2 x 10^3^ cells/well for HCT-116, and 1 x 10^4^ cells/well for NCM460. Cells were treated with the studied compound dissolved in PBS with a final concentration of DMSO less than 0.2% (v/v). Following treatment, the WST-1 molecule was added to each well and incubated for approximately four hours. Absorbance readings at 450 nm were recorded using a SpectraMax Gemini XS multi-well plate reader (Molecular Devices, Sunnyvale, CA, USA) and expressed as a percentage of the DMSO control.

### Quantification or early and late apoptosis

Annexin V binding, a marker of early apoptosis, was used to monitor apoptosis [29]. In parallel, propidium iodide (PI) uptake was monitored as a marker of cell permeabilization and late apoptotic death. Cells were seeded in six-well plates and grown for an additional 24 hours. Cells were treated with either DMSO vehicle or the studied compound for either 6, 12, 24, or 48 hours. Cells were collected, washed with PBS, and resuspended in Annexin V binding buffer (10 mM HEPES, 140 mM NaCl, 2.5 mM CaCl_2_, pH 7.4), and incubated with Annexin V AlexaFluor-488 (1:20; Life Technologies Inc, Cat. No. A13201, Burlington, ON, Canada) and propidium iodide (0.01 mg/mL; Life Technologies Inc, Cat. No. P3566, Burlington, ON, Canada) for 15 minutes at 37°C protected from light. Following incubation, the percentage of early and late apoptotic cells were quantified using image-based cytometry with a Tali® Image-Based Cytometer (Life Technologies Inc, Cat. No. T10796, Burlington, ON, Canada). Cells from at least 18 fields were analyzed using both the green (ex. 458 nm; em. 525/20 nm) and red (ex. 530 nm; em. 585 nm) channels. Staurosporine (STS; Sigma-Aldrich Canada, Cat. No. S4400, Mississauga, ON, Canada) is a known inducer of apoptosis that was utilized as a positive control.

### Differential Interference contrast microscopy of cells grown in 3D culture

Cells were plated in round-bottomed, ultra-low adherence plates (Corning Inc, Cat No. 7007, Corning, NY), incubated for 24 hours, and treated with vehicle control or the studied compounds. Following treatment, cells were incubated for an additional 72 hours before being analyzed via differential interference contrast (DIC) microscopy using a Leica DM IRB inverted fluorescence microscope (Leica Microsystems, Wetzlar, Germany) at 10× objective lens.

### Cell cycle analysis

MV-4-11 cells were seeded in 60 mm x 15 mm plates and allowed to grow for 48 hours before treatment with either DMSO vehicle or studied compound. Each group had the same final DMSO concentration. Treatment progressed for 3, 6, 12, or 24 hours and the cells were collected and washed once with PBS. Approximately 1, 000, 000 cells from each group were fixed using ice cold 80% ethanol. Cells were incubated for at least 24 hours at -20°C, washed with ice cold PBS, and incubated in a PI staining solution (0.1% Triton® X-100 (Sigma-Aldrich Canada, Cat. No. T8787, Mississauga, ON, Canada), 0.2 mg/mL RNase A (Sigma-Aldrich Canada, Cat. No. R6513, Mississauga, ON, Canada), 20 μg/mL PI) for 20 minutes at room temperature protected from light. The amount of PI fluorescence per cell, correlating to DNA content, was quantified using image-based cytometry with a Tali® Image-Based Cytometer (Life Technologies Inc, Cat. No. T10796, Burlington, ON, Canada). Cells from 20 fields were analyzed using the red (ex. 530 nm; em. 585 nm) channel. Data was exported in the fcs format and analyzed using FCS Express 4 (flow research edition). Using the sliced background fit model, the program provided a fit based on the raw data and calculated the percentage of cells in the G_1_/G_0_ phase, S phase, and G_2_/M phase.

### Quantification of mitochondrial membrane potential (MMP)

Tetramethylrhodamine methyl ester (TMRM) staining was conducted to monitor mitochondrial membrane potential (MMP). TMRM accumulates within intact mitochondria due to mitochondrial polarization, followed by a red shift in fluorescence emission spectra. Loss of red fluorescence indicates MMP dissipation. Cells were seeded in six-well plates, grown for 24 hours, and treated with DMSO vehicle or the studied compound. Each group had the same final DMSO concentration. Treatment was allowed to progress for 3, 6, 12, 24, or 48 hours. Cells were collected, washed with PBS, and resuspended in fresh media. Cells were incubated in 10 nM TMRM (Gibco BRL, VWR, Mississauga, ON, Canada) for 45 minutes at 37°C protected from light. Following incubation, cells were washed with PBS and percentage of cells with intact MMP was quantified using image-based cytometry with a Tali® Image-Based Cytometer (Life Technologies Inc, Cat. No. T10796, Burlington, ON, Canada). Cells from at least 18 fields were analyzed using the red (ex. 530 nm; em. 585 nm) channel. Taxol (Sigma-Aldrich Canada, Cat. No. T7402, Mississauga, ON, Canada) and pancratistatin (PST; obtained from Dr. McNulty) were used as positive controls for mitochondrial membrane permeabilization.

### Western blotting

MV-4-11 and E6-1 cells were treated with either negative control (DMSO vehicle) or studied compound dissolved in DMSO. Cells were collected, washed with PBS, and lysed using NP40 lysis buffer (0.1% NP40, 20 mM Tris HCL, 100 mM NaCl, 5 mM EDTA, 10 μM Leu-pep, 10 μM Pep-A, and 100 μM PMSF). SDS-PAGE was performed on the protein samples. 40 μg of protein was loaded per well. Electrophoresed proteins were transferred to a PVDF membrane, following which, membranes were blocked with a 5% w/v milk TBST solution for 1 hour. After being blocked, membranes were probed with various primary antibodies overnight at 4°C: cyclin B1 raised in rabbit (1:1500; Novus Biologicals, Cat. No. NBP1-61243, Littleton, CO, USA) and β-Actin raised in mouse (1:2000; Santa Cruz Biotechnology, Inc., Cat. No. sc-81178, Paso Robles, CA, USA). Following primary antibody incubation, membranes were washed and incubated with a goat anti-mouse (1:3000; Novus Biologicals, Cat. No. NBP2-30347H, Paso Robles, CA, USA) or a goat anti-rabbit (1:3000; Novus Biologicals, Cat. No. NBP2-30348H) horseradish peroxidase-conjugated secondary antibody for 1 hour at 4°C. Bands were visualized with enhanced chemiluminescence reagent (Sigma-Aldrich Canada, Cat. No. CPS160, Mississauga, ON, Canada) and densitometry analyses were performed using ImageJ software.

### Computational analysis

The protein crystal structure of tubulin docked with colchicine (PDB code: 1SA0) was downloaded from RCSB protein data bank. The structure was loaded into Molecular Operating Environment (MOE), which was used to simulate docking of CA4, MP5-F9, and MP5-G9 to the colchicine binding pocket. Using the Protonate3D algorithm, hydrogen atoms were added to the protein structure. The water molecules present in the crystal structure were deleted and the protein was resolvated with water molecules in a more optimal position. The entire protein system was minimized using the AMBER12:EHT force field to relax the protein in a solvated environment. Prior to docking of CA4, MP5-F9, or MP5-G9, the bound-colchicine was deleted from the binding site. To find the optimal binding orientation of the three compounds, an initial docking procedure using the first coordination sphere was conducted. The proxy triangle algorithm scored numerous structures (up to 500 unique structures), which were then placed in the active site and scored by the London dg scoring function. The top 50 poses for each compound were energy minimized in the active site, before being scored once again using the London dg scoring function, after which the first set of docked ligands was elucidated. Using the determined poses of CA4, MP5-F9, and MP5-G9 docked in the active site, a molecular dynamic simulation was conducted as to allow the system to find a local minimum over 5 nanoseconds. After identifying the protein system with minimal energy, the poses identified from the first docking studies were re-docked to determine the free-energy of binding and the affinity of the ligand for the binding site.

### Statistics

All experiments contained multiple variables (including various doses, drugs, and time points) and were analyzed via two-way ANOVA statistical analysis. In instances where experimental groups were compared to the control, the Dunnet post hoc test was used to determine significance. If comparisons were being made between various experimental groups as well as the control group, then a Tukey post hoc test was used. IC_50_ values were determined through interpolation using non-linear regression. All statistical analysis was conducted using GraphPad Prism 6 statistical software. A *p*-value below 0.05 was considered significant.

## Results

### Evaluation of anti-proliferative and cytotoxic properties of CA4 and biphenyl analogues

**Fig 2:**
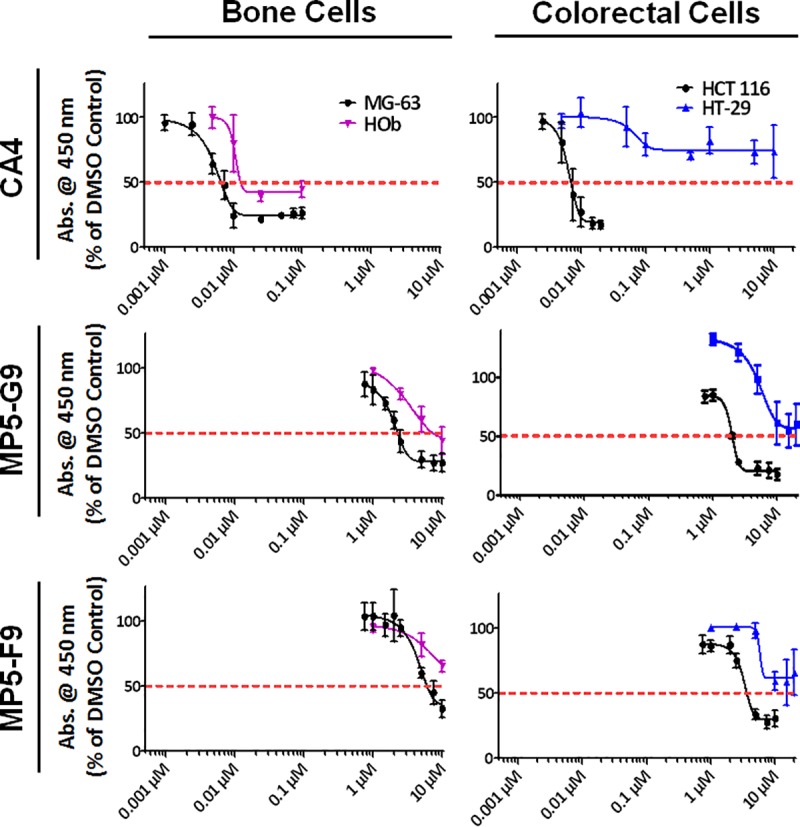
CA4 and biphenyl analogues inhibit proliferation of cancer cells. Various cancerous and non-cancerous cells were treated with the indicated drug at the indicated dose for 48 hours and the WST-1 reagent was used to quantify proliferation. Absorbance was read at 450 nm and expressed as a % of DMSO control. MG-63 = osteocarcoma cells; HCT-116, HT-29 = colorectal cancer; HOb = normal human osteoblasts. Values are expressed as mean ± SD from quadruplicates of at least three independent experiments.

CA4 was found to have potent anti-proliferative activity in an osteocarcoma cell line (MG-63) and one of two colorectal cancer cell lines (HCT-116, HT-29; Fig 2). Additionally, treatment with CA4 was also found to inhibit proliferation of non-cancerous human osteoblast (HOb) cells in a dose-dependent manner. The one cell line that was only minimally affected by treatment with CA4 was the HT-29 colorectal cancer cell line, which had been previously established as CA4-resistent [30]. Biphenyl analogues of CA4, MP5-F9 and MP5-G9, were also evaluated and were determined to have a similar ability to inhibit cancer proliferation. However, the dose required to reduce cancer proliferation was two to three orders of magnitude more concentrated than what was required by CA4 to have a similar effect. The half-maximal inhibitory concentrations (IC_50_), as determined by non-linear regression, are summarized in Table 1. These findings are a continuation of a previously published study in which preliminary characterization of MP5-G9 and MP5-F9 was reported [18](18). In that study, we reported that CA4 and the two biphenyl analogues possessed anti-proliferative and pro-apoptotic activity in two additional osteocarcoma cell lines (Saos-2, U-2 OS), and two leukemia cell lines (E6-1, MV-4-11).

**Table 1:** IC_50_ values of compounds MP5-F9, MP5-G9, and CA4 in various cancerous cell lines.

	IC_50_ (μM)		
Cell Line	CA4	MP5-F9	MP5-G9
MG-63	0.00767 ± 0.00046	6.16 ± 0.40	2.29 ± 0.21
HOb	~0.0124	> 10	~7.79
HCT-116	0.00689 ± 0.0015	3.57 ± 0.48	2.01 ± 0.034
HT-29	> 10	> 20	>20

IC_50_ values were determined at the 48 hour time point in several cancerous and non-cancerous cell lines. The IC_50_ values were determined through interpolation using non-linear regression software. Values are expressed as mean ± SD of at least three trials. For HOb experiments with CA4 and MP5-G9, an IC_50_ value for each trial could not be obtained. Instead, an IC_50_ value for the average of three trials was determined.

**Fig 3:**
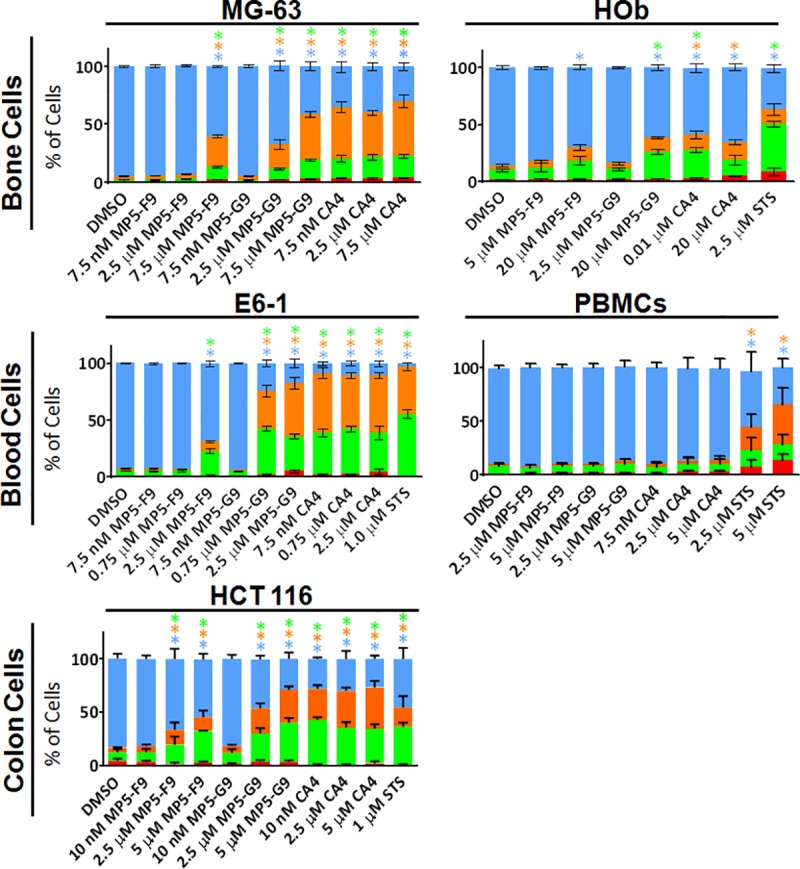
CA4 and biphenyl analogues induce apoptosis in cancer cells. Various cancerous and non-cancerous cells were treated with the indicated drug at the indicated dose for 48 hours. Cells were then probed with annexin V (green fluorescent probe that binds to apoptotic cells) and propidium iodide (PI), a red fluorescent probe that binds to cells with permeabalized membranes. Percentage of annexin V and PI positive cells was quantified using image-based cytometry. Blue bar represents viable cells; orange bar represents PI and annexin V positive cells; green bar represents annexin V positive cells; red bar represents PI positive cells. Staurosporine (STS) was utilized as a positive control. Values are expressed as mean ± SD from at least three independent experiments. * *p* < 0.05 vs. DMSO control.

To determine if treated cells were indeed undergoing apoptosis, cells were probed with annexin V, conjugated to alexa fluor 488, and propidium iodide (PI). Early during apoptosis, the cell membrane is reorganized, which allows for annexin V binding and detection. PI can only enter the cell, intercalate with DNA, and fluoresce red when the cell membrane has been compromised, which occurs during late apoptosis. Cells positive for only annexin V were considered to being undergoing early apoptosis, while cells positive for both annexin V and PI were considered to be undergoing late apoptosis or secondary necrosis. Cells that were not successfully probed with either PI or annexin V were considered viable. At the 48 hour time point, it was seen that CA4 and analogues possessed the ability to induce apoptosis in a variety of cancer cell lines (Fig 3). The highest tested doses of compound MP5-G9, which ranged from 2.5 to 7.5 μM, yielded apoptotic indexes similar to that of CA4, although at doses that were considerably more concentrated.

In addition to the cancer cell lines tested, various non-cancerous equivalents were utilized to evaluate the selectivity of CA4 and its analogues. Although non-cancerous HOb cells experienced a dose-dependent decrease in proliferation following treatment with CA4 and analogues, treated HOb cells underwent less apoptosis, when compared to cancerous osteoblast cells (Fig 3). Likewise, non-cancerous human peripheral blood mononuclear cells (PBMNCs) were resistant to the pro-apoptotic outcomes associated with treatment with CA4 and analogues (Fig 3). We previously reported that NCM460 normal colon mucosa cells are resistant to treatment with CA4 and biphenyl analogues[18]. Interestingly, HT-29 cells were also resistant to treatment with CA4 and analogues (Fig 2). Speculatively, it is tempting to suggest that these cell lines may share a common drug resistant pathway. However, the difference in response to treatment may be the result of differing proliferation rates, which was not quantified.

**Fig 4:**
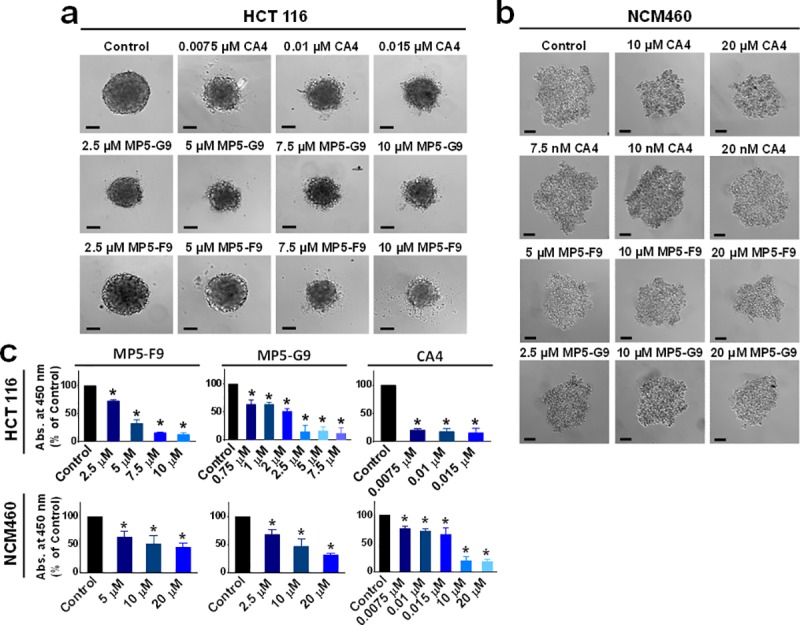
CA4 and biphenyl analogues reduce growth and proliferation of colorectal cancer spheroids selectively. (A) HCT116 cancer cells and (B) NCM460 normal colon mucosa cells were grown in ultra-low adherent round-bottomed 96-well plates for 48 hours to establish spheroid formation. Subsequently, spheroids were treated with indicated dose of either CA4 or biphenyl derivative for 72 hours. Spheroid morphology and size was monitored via differential interference contrast microscopy. Scale bar = 500 microns. (C) The WST-1 reagent was used to quantify proliferation. Absorbance was read at 450 nm and expressed as a % of DMSO control. * *p* < 0.05.

It has been shown that the cancer microenvironment and tumour architecture can dictate the sensitivity of cancer to various cytotoxic agents. To better approximate what occurs in the human body, the previously tested HCT 116 colorectal cancer cells and NCM460 normal colon mucosa cells were grown in 3D culture, using low-adherent plates with rounded bottoms, which promoted spheroid formation (Fig 4). Following 72 hour treatment with either CA4 or biphenyl derivative, HCT 116 spheroid size was diminished when compared to the vehicle control treated group (Fig 4A). Similar experiments were conducted with Saos-2 osteocarcoma cells grown in 3D culture and a comparable effect on spheroid size was observed (S1 Fig). In addition, the proliferation of HCT 116 cells grown in 3D culture, as quantified via a WST-1 cell proliferation assay, was also found to be significantly decreased following treatment with either CA4 or analogues (Fig 4C). NCM460 cells formed looser spheroids, which were less adversely effected by treatment with either CA4 or biphenyl analogues (Fig 4B and 4C). These observations suggested that biphenyl analogues of CA4 can inhibit growth of colorectal cancer cell spheroids.

### Cell cycle arrest precedes mitochondrial collapse, DNA damage, and apoptotic induction associated with CA4-mediated cell death

Mechanistic studies were conducted to complement the cytotoxic evaluation of CA4 and analogues. It has been generally accepted that CA4 induces apoptosis in cancer cells *in vitro* through a mechanism dependant on the disruption of microtubule dynamism and prolonged activation of mitotic checkpoints [19, 21]. However, as discussed above, there are several lines of evidence that introduce uncertainty surrounding this hypothesis, including the failure of various small-molecule inhibitors to rescue CA4-treated cells from death and the existence of cytotoxic CA4-analogues that do not appear to target microtubules [19, 20, 27].

**Fig 5:**
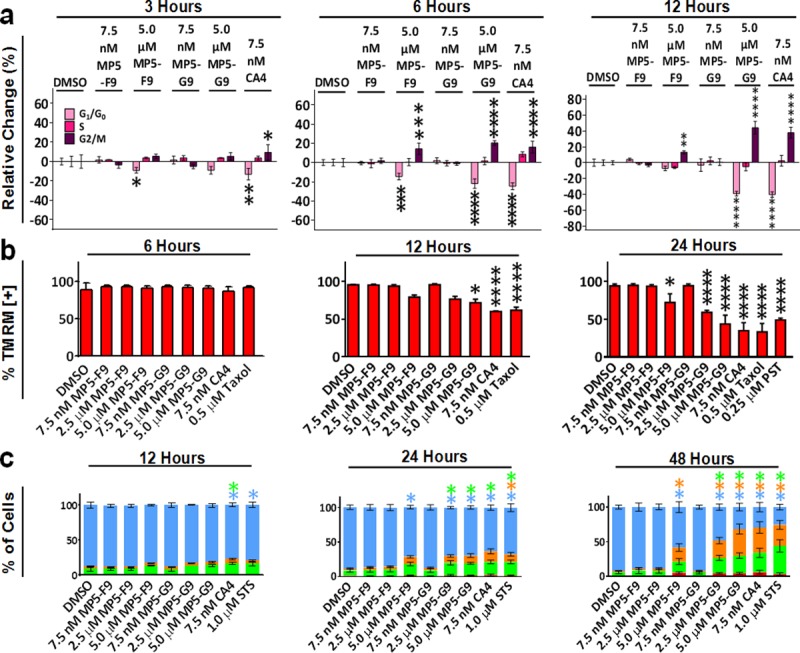
CA4 and biphenyl analogues induced G2/M arrest precedes mitochondrial collapse and apoptotic induction. (A) Treated cells (dose and time are indicated) were fixed overnight with ice cold 80% ethanol and stained with propidium iodide. Cell cycle profiles were generated using image-based cytometry. Values are expressed as mean ± SD of three independent experiments. * *p* < 0.05 versus DMSO control; *** *p* < 0.001 versus DMSO control; **** *p* < 0.0001 versus DMSO control. (B) Treated cells were probed with tetramethylrhodamine methyl ester (TMRM). Taxol and pancratistatin (PST) treated cells served as positive controls. Percentage of TMRM positive cells was quantified using image-based cytometry. Values are expressed as mean ± SD of three independent experiments. * *p* < 0.05 versus DMSO control; **** *p* < 0.0001 versus DMSO control. (C) Treated cells were probed with annexin V and propidium iodide (PI). Percentage of annexin V and PI positive cells was quantified using image-based cytometry. 1.0 μM staurosporine (STS) treated cells served as a positive control. Blue bar represents viable cells; orange bar represents PI and annexin V positive cells; green bar represents annexin V positive cells; red bar represents PI positive cells. Values are expressed as mean ± SD from three independent experiments. * *p* < 0.05 vs. DMSO control.

To evaluate the importance of prolonged mitotic arrest for CA4-mediated cell death, a chronology of cellular events that occur following CA4 treatment was established with the MV-4-11 chronic myelomonocytic leukemia cell line (Fig 5). It was seen that G2/M cell cycle arrest was induced by CA4 and analogues as early as 3 hours post treatment, and peaked at the 12 hour time point (Fig 5A). The increase in G2/M phase was accompanied by a similar decrease in G1 phase, with little to no difference in the number of cells in the S phase. Mitochondrial depolarisation became evident at the 12 hour time point and progressed in a time-dependent manner (Fig 5B). Mitochondrial depolarisation was not seen at either the 3 hour or 6 hour time point (S2 Fig, Fig 5B). Phosphatidylserine externalization, a marker of early stage apoptosis, was witnessed at the 24 hour time point using an annexin V binding assay and was accompanied with very little cell membrane permeabilization, indicated by minimal PI staining (Fig 5C). There was little to no apoptotic induction or membrane permeabilization at earlier time points (S2 Fig, Fig 5C). However, at the 48 hour time point, there was widespread apoptosis and a large number of cells had permeabilized (Fig 5C). Thus, the chronological G2/M arrest, mitochondrial depolarisation, phosphatidylserine externalization, and membrane permeabilization suggested that CA4 and biphenyl analogues induced apoptosis via a mechanism that was dependent on prolonged mitotic arrest. Based on the cell cycle analysis, which also gave a measure of the number of cells with DNA damage, it was seen that a sizeable subG0 population was not present until the 12 hour time point, at which point there was already significant G2/M arrest (S3 Fig). This observation suggests that G2/M arrest was not the result of DNA damage but direct microtubule targeting.

### Small molecule inhibition of mitotic arrest abolishes CA4-mediated cytotoxicity

**Fig 6:**
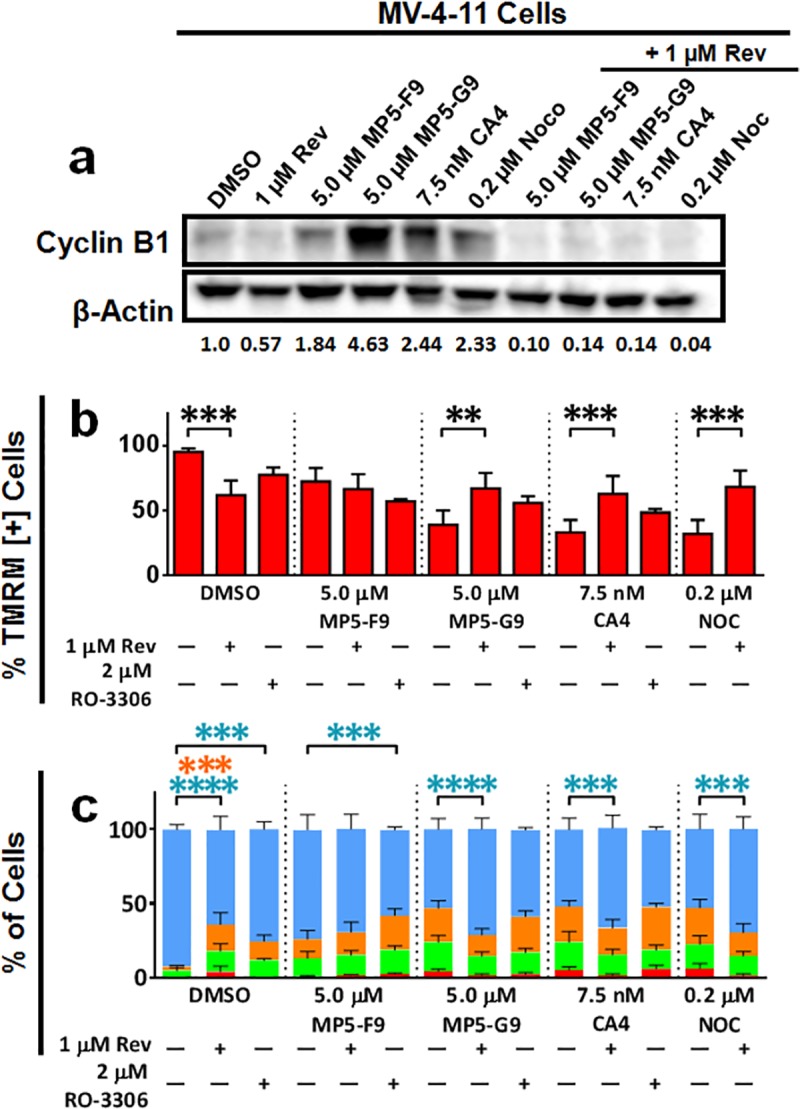
Small molecule inhibitors of mitotic arrest able to ameliorate mitotic arrest, mitochondrial collapse, and apoptotic induction. MV-4-11 cells were treated with either CA4, MP5-F9, MP5-G9, or nocodazole (NOC), alone or co-incubated with either reversine (rev) or RO-3306, for 24 hours. (A) Whole cell lysates were electrophoresed, transferred to a PVDF membrane and probed for cyclin B1 and β-actin. Bands were visualized with enhanced chemiluminescence reagent and densitometry analyses were performed using ImageJ software. Values are expressed as the ratio cyclin B1 band intensity over β-actin band intensity and are normalized to DMSO control. Western blot images are representative of three independent trials. (B) Treated cells were probed with tetramethylrhodamine methyl ester (TMRM). Percentage of TMRM positive cells was quantified using image-based cytometry. Values are expressed as mean ± SD of at least three independent experiments. ** *p* < 0.01; *** *p* < 0.001. (C) Treated cells were probed with green fluorescent annexin V and propidium iodide (PI), a red fluorescent probe. Percentage of annexin V and PI positive cells was quantified using image-based cytometry. Blue bar represents viable cells; orange bar represents PI and annexin V positive cells; green bar represents annexin V positive cells; red bar represents PI positive cells. Values are expressed as mean ± SD from at least three independent experiments. *** *p* < 0.001; **** *p* < 0.0001.

To further evaluate the necessity of mitotic arrest for CA4-mediated cytotoxicity, small molecule inhibitors reversine and RO-3306 were utilized. Reversine is a specific MPS1 kinase inhibitor [31]. MPS1 is a component of the spindle checkpoint and has been shown to be required for the maintenance of the mitotic checkpoint [32, 33]. RO-3306 is a specific inhibitor of cyclin dependent kinase-1 (CDK1), which is required for signaling the G2-M transition [34]. In MV-4-11 leukemia cells, co-incubation of reversine with CA4 and analogues concomitantly prevented mitotic arrest, as evidenced by cyclin B1 western blotting (Fig 6A), and mitochondrial depolarisation (Fig 6B), which suggested a causal connection. Similarly, RO-3306 was also able to prevent mitochondrial depolarisation (Fig 6B). Additionally, both RO-3306 and reversine were able to rescue cells treated with either CA4 or biphenyl analogues from apoptosis, suggesting that apoptosis was also dependent on prolonged mitotic arrest (Fig 6C). Similar results were witnessed in the E6-1 leukemia cell line (S4 Fig). Importantly, in E6-1 cells, RO-3306 treatment alone was shown to induce a modest accumulation of cyclin B1, suggesting that the inhibitor was able to promote G2/M arrest (S4 Fig). Furthermore, co-treatment of cells with RO-3306 moderated the accumulation of cyclin B1 induced by CA4 and analogues (S4 Fig), suggesting that the inhibitor was inducing G2 blockade, as cyclin B1 levels are more prominent during mitosis than during G2 phase [35].

For the inhibition studies, nocodazole (NOC) was utilized as a positive control, as a previous study had shown that staurosporine, a general serine/threonine kinase inhibitor, was able to ablate both mitotic arrest and apoptosis in nocodazole treated cells [36]. In our study, reversine, a specific kinase inhibitor, was also able to concomitantly prevent the mitotic arrest, mitochondrial depolarisation, and apoptosis normally associated with nocodazole (Fig 6A–6C, S4 Fig).

### Abolishment of the extrinsic pathway of apoptosis delays CA4-mediated cytotoxicity

**Fig 7:**
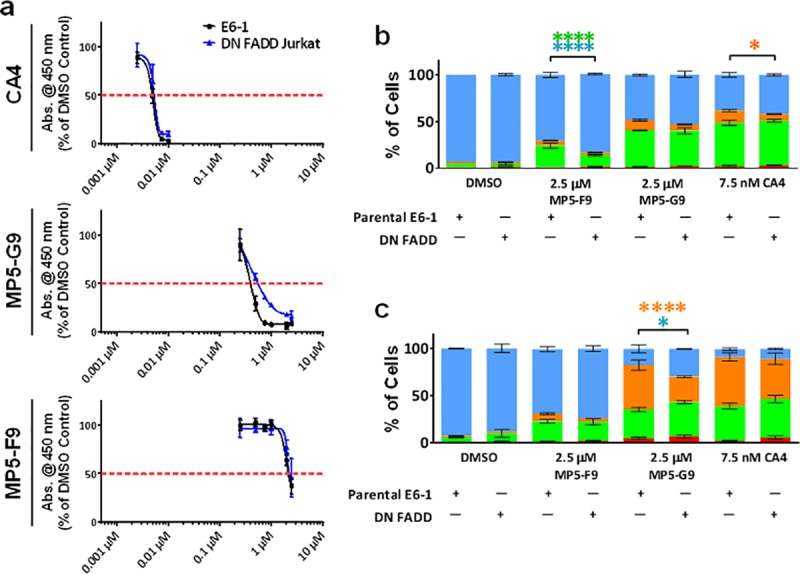
Cells with defective extrinsic pathway of apoptosis experience delayed response to treatment with CA4 and biphenyl analogues. **(**A) Wild-type E6-1 leukemia cells and dominant-negative Fas-associated via death domain (DN FADD) leukemia cells were treated with the indicated drug at the indicated dose for 48 hours and the WST-1 reagent was used to quantify proliferation. Absorbance was read at 450 nm and expressed as a % of DMSO control. (B, C) Cells were treated for either (B) 24 hours or (C) 48 hours and were probed with annexin V and propidium iodide (PI). Percentage of annexin V and PI positive cells was quantified using image-based cytometry. Blue bar represents viable cells; orange bar represents PI and annexin V positive cells; green bar represents annexin V positive cells; red bar represents PI positive cells. Values are expressed as mean ± SD from three independent experiments. * *p* < 0.05 vs. DMSO control; **** *p* < 0.0001 vs. DMSO control.

Previous work has shown that several CA4 analogues can activate the extrinsic pathway of apoptosis [23, 27]. For certain analogues, there was even a partial dependency on the cell death pathway for eliciting a cytotoxic effect, which was evidence for CA4 potentially having additional cellular targets [23]. Dominant-negative Fas-associated via death domain (FADD) jurkat cells were utilized as a cell line defective for the extrinsic pathway of apoptosis [37]. At the 48 hour time point, treatment with either MP5-F9 or CA4 yielded a similar anti-proliferative profile in both wild-type E6-1 jurkat cells and DN-FADD jurkat cells (Fig 7A). However, compound MP5-G9 had a diminished anti-proliferative effect in DN-FADD cells. Annexin V and PI probing revealed that CA4 treatment resulted in a significantly reduced population of late apoptotic cells in DN-FADD cells following 24 hours, and MP5-F9 treatment resulted in significantly fewer early apoptotic cells in DN-FADD cells at the same time point (Fig 7B). At the 48 hour time point, there were significantly fewer late apoptotic cells in DN-FADD cells treated with compound MP5-G9, as compared to E6-1 cells, which correlated with the WST-1 experiments at the same time point (Fig 7C).

### Predicted tubulin affinity correlates with cytotoxicity of CA4 and biphenyl analogues

**Fig 8:**
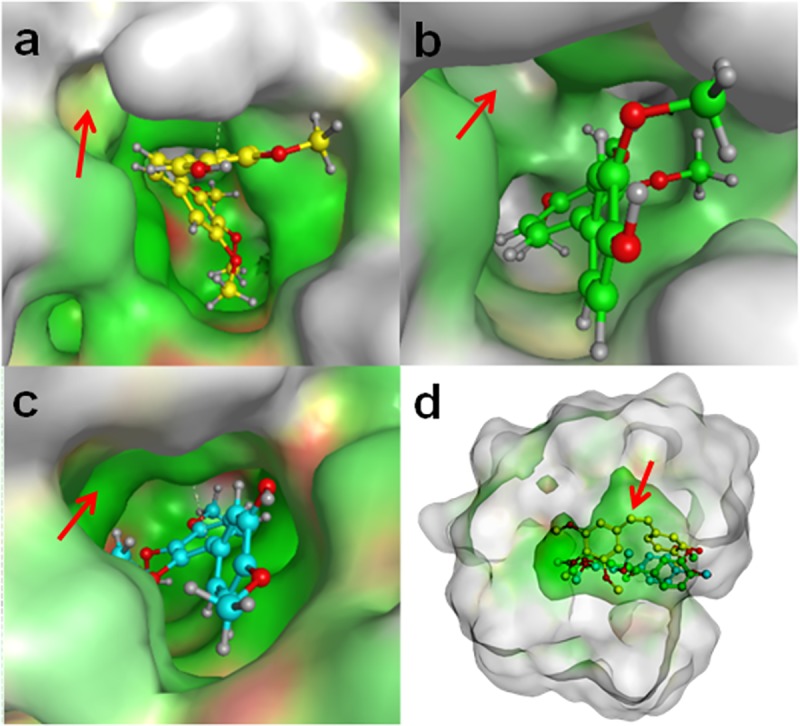
Predicted Tubulin Affinity Correlates with Cytotoxicity of CA4 and Biphenyl Analogues. Green = hydrophobic residues; red = polar residues; white = non-interacting residues. Using Molecular Operating Environment (MOE) software, CA4, MP5-F9, and MP5-G9 were separately docked in the colchicine binding pocket, whose crystal structure was downloaded from the protein data bank (PDB code: 1SA0). Red arrow indicates the same hydrophobic pocket within the overall colchicine binding site, which is occupied by the ethylene linker of CA4. (A) CA4 (B) MP5-G9 (C) MP5-F9 (D) Merged image of CA4 and CA4 analogues docked to colchicine binding site. Yellow = *cis*-CA4; Green = MP5-G9; Blue = MP5-F9.

Structure-activity relationship (SAR) studies have revealed that the 3,4,5-trimethoxyphenyl group of ring A (Fig 1) is essential for activity and that the 4-methoxy group of ring B (Fig 1) is beneficial for cytotoxicity, while the 3-hydroxy group is not essential [6]. Additionally, the *cis* configuration about the ethylene linker has been shown to be crucial for CA4 activity, as corresponding *trans-*stilbenes retain very little activity [38]. The role of the ethylene linker has been speculated to be largely involved with the appropriate spacing and angling of the two aromatic rings [6]. To evaluate the role of the ethylene linker further, we conducted *in silico* docking experiments of CA4 and its biphenyl analogues to the tubulin heterodimer, whose crystal structure was solved in complex with a colchicine analogue (PDB: 1SAO; Fig 8A–8D).

The free energy of binding for CA4, MP5-F9, and MP5-G9 revealed a trend that supported the hypothesis that the ethylene linker is important for increasing the binding ability of colchicinoids (Table 2). CA4 was computationally predicted to bind tubulin spontaneously with a free energy of -81.4 kJ/mol, whereas MP5-F9 and MP5-G9 were predicted to bind with a free energy of -59.2 kJ/mol and -58.1 kJ/mol, respectively. The trend revealed that predicted CA4 binding was more favourable than either MP5-F9 and MP5-G9, which bound with similar free energy. Thus, predicted tubulin affinity correlated with the presence of an ethylene linker and cytotoxicity towards cancer cells.

**Table 2:** CA4 and biphenyl analogues are predicted to bind favourably to the colchicine binding site of tubulin.

	Binding Affinity (kcal/mol)	Free Energy (kJ/mol)	Relative Free Energy (compared to CA4; kJ/mol)
**MP5-F9**	-14.2 ± 0.76	-59.2 ± 3.1	22.2
**MP5-G9**	-13.9 ± 0.44	-58.1 ± 1.8	23.3
**CA4**	-19.5 ± 1.0	-81.4 ± 4.2	0

The binding affinity and free energy of binding associated with MP5-F9, MP5-G9, and CA4 binding to the colchicine binding site of tubulin was computationally determined. Using Molecular Operating Environment software, the compounds were docked in the colchicine binding site of tubulin, following which molecular dynamic simulation took place to relax the structure. Compounds were re-docked and associated binding affinity and free energy was determined using the London dG scoring function. Values are representative of mean ± SD of three random stationary points of the average cluster of tubulin protein docked with studied compound.

## Discussion

A major component of this research was dedicated towards screening the anti-proliferative and pro-apoptotic activity of novel biphenyl analogues in 2D cancer cell models. The detailed *in vitro* characterization serves as a proof-of-concept and suggests that biphenyl analogues of CA4 can retain modest anti-proliferative and pro-apoptotic properties. It was found that MP5-F9 and MP5-G9, in this study (Fig 2) and our previously published work [18], were able to reduce cellular proliferation, below 50% relative to the vehicle treated control, in two of three osteocarcoma, one of two colorectal cancer, and both leukemia cell lines tested. Those results were supported by annexin V and PI probing, which revealed that cancer cells treated with MP5-F9 and MP5-G9 underwent apoptosis (Fig 3). Thus, evidence has been obtained to show that structurally simplified analogues of CA4 can indeed possess anti-proliferative and pro-apoptotic activity. These results are exciting because multiple biphenyl CA4 analogues (S5 Fig) have been previously characterized and were found to be devoid of anti-cancer and anti-microtubule activity [39, 40]. The inactive biphenyl analogues feature the crucial trimethoxy moiety on ring A, but an offset positioning of the methoxy groups when compared to CA4. As the importance of the trimethoxy moiety has been well-established, it makes sense that previous examples of biphenyl CA4 analogues were inactive while the currently studied analogues possess some degree of activity and contain a trimethoxy moiety that is positioned in a manner similar to CA4. Previously, 2-methoxy-5-(2', 3', 4'-trimethoxyphenyl)tropone (AC; S5 Fig), a biaryl analogue of colchicine lacking the adjoining the seven-membered ring but possessing the appropriate trimethoxy moiety, has also been found to be a potent MTA, further validating the therapeutic potential of structurally-simplified analogues of CA4 and colchicine [39].

*In silico* docking studies of *cis*-CA4 and the two novel biphenyl analogues were conducted to further explore the role of the ethylene linker in CA4 binding to tubulin (Fig 8). Interestingly, CA4 was predicted to bind more favourably to the colchicine site on tubulin, when compared to biphenyl analogues that lack an ethylene linker. Thus, it appears that the presence of an ethylene linker is beneficial for tubulin binding and explains why the biphenyl analogues are less cytotoxic. This is consistent with what has been reported in the literature. Previous computational modeling of CA4 and analogues has revealed that predicted tubulin affinity correlates with *in vitro* cytotoxicity [41–43].

In addition to performing a SAR study, we also sought to further evaluate the mechanism behind CA4's *in vitro* activity. It has been previously suggested that MTAs specifically bind to tubulin and have few other direct cellular targets [44]. However, recent studies have uncovered additional targets of well-studied tubulin binding agents. Paclitaxel has been shown to bind directly to Bcl-2, which results in the opening of the mitochondrial permeability transition pore [45]. Such recent revelations, in addition to the observation that multiple CA4 analogues possess anti-cancer activity while displaying little to no anti-microtubule activity, prompted our hypothesis that CA4 may possess additional cellular targets that are implicated with its pronounced cytotoxicity towards cells *in vitro*.

To first test this hypothesis, we completed a chronology of cellular events that occur following treatment of MV-4-11 leukemia cells with CA4 and analogues. It was observed that cells were arrested in G2/M at early time points (3 to 12 hours), which preceded mitochondrial depolarisation (12 to 24 hours), phosphatidylserine externalization and membrane permeability (24 to 48 hours). Although these cellular events have been previously identified amongst cells treated with CA4, this experimentation represents the first attempt to determine which events occur first and may be causative of the other events. The ordering of the events lends strength to the idea that prolonged G2/M arrest is the main response of cells to CA4 treatment, which in turn triggers other cellular responses, such as mitochondrial depolarisation and apoptotic induction.

To further test our hypothesis, we utilized the small molecule inhibitor reversine, which inhibits MPS1 kinase activity, crucial for the spindle checkpoint and its ability to maintain mitotic arrest. It was found that treatment groups that received both CA4 and reversine experienced less mitochondrial depolarisation and apoptotic induction, which was concomitant with decreased levels of cyclin B1, a marker of mitosis. Similar observations were made when cells were treated with both MP5-G9 and reversine, suggesting similar cell death pathways are activated. Our results with CA4 are similar to that seen with the MTA nocodazole. When co-incubated with staurosporine, which was shown to prevent mitotic arrest at low doses, a similar cytoprotective benefit was witnessed, pointing to the necessity of prolonged mitotic arrest for nocodazole-induced apoptosis [36]. Importantly, our studies show that reversine could similarly prevent the apoptosis, as well as mitochondrial depolarisation, associated with nocodazole.

A similar trend was noticed when CA4 was co-incubated with the selective CDK1 inhibitor, RO-3306. RO-3306 could prevent CA4-linked mitochondrial depolarisation in both leukemia models tested, although the protective effect was only significant in the E6-1 cell line. In addition, RO-3306 could prevent a decrease in cell viability normally associated with CA4 treatment, in the E6-1 leukemia cell line. Our results are consistent with previously published data. It had been previously shown that pharmacological inhibition of p34^cdc2^ prevented both mitotic arrest and a decrease in cellular viability in CA4P-treated endothelial cells [35]. Overall, we obtained results using two independent small molecule inhibitors, which strongly suggest that CA4 functions primarily as a MTA to kill cancer cells *in vitro*.

Despite chemical ablation of mitotic arrest being able to rescue cancer cells, we have uncovered preliminary evidence that the extrinsic pathway of apoptosis may also play a role in CA4- linked cell death. We have uncovered that cells DN-FADD jurkat cells experience delayed cell death when compared to jurkat cells not expressing dominant negative FADD protein. This observation is consistent with emerging evidence that CA4 can trigger an increase in Fas levels and pro-caspase eight cleavage, which are markers of the extrinsic pathway of apoptosis [37] and that the use of a Fas-blocking monoclonal antibody can rescue cancer cells from treatment with certain CA4 analogues [23]. The degree to which this pathway is significant for CA4-mediated cell death warrants further investigation.

To date, research has found that the activation of multiple cellular pathways associated with cell death can be causally linked to CA4's microtubule targeting activity. A study has shown that Bim, a pro-apoptotic protein, is released from its interaction with dynein, a motor protein associated with microtubules, following CA4 treatment and facilitates the depolarisation of the mitochondria [46]. Similarly, p53 has been shown to be released from the microtubule network, translocate to the mitochondria, and interact with Bcl-2 and Bim, following CA4-mediated microtubule disruption [47]. Additionally, activation of the GTPase Rho by CA4 and analogues has been linked to microtubule disruption and shown to induce re-organization of the actin cytoskeleton, including increased actin polymerization, actinomyosin contractility, assembly of actin stress fibres, and the formation of focal adhesions in endothelial cells, which has been put forth as an explanation for CA4’s *in vivo* vascular damaging activity [48, 49]. Thus, microtubule targeting activity has been implicated in a wide range of cellular responses to CA4 treatment.

However, as discussed in the introduction, previous attempts to identify which cellular targets are activated following CA4 treatment and are crucial for cytotoxicity have failed to identify a single cell death pathway. The use of PARP and caspase inhibitors have failed on separate occasions to save cells from dying, despite ample evidence suggesting that caspases are activated and AIF is released from the mitochondria, following CA4 treatment. Although this discrepancy is a source of confusion, it is possible that since CA4-mediated mitochondrial depolarisation results in both caspase activation and AIF release, the use of a single inhibitor is unable to rescue cells from death. Additionally, cytochrome c has also been shown to be released from the mitochondria of cells treated with CA4, which furthers the complexity [21]. Potentially, the use of caspase inhibitors in combination with cytochrome c knockdown and PARP inhibitors may yield cell rescue and a more conclusive implication of the mitochondria as a major player during CA4-linked cell death.

Our study has characterized two structurally-simplified analogues of C4, which possess anti-proliferative and pro-apoptotic properties in various cancer cell lines, both in 2D and 3D culture models. The biphenyl derivatives, as well as CA4, have been shown to induce mitotic arrest, which has been identified as necessary for potent pro-apoptotic activity. Lastly, computational analysis has revealed that predicted tubulin affinity correlates with the presence of an adjoining ethylene linker, which explains the more potent activity of CA4 and analogues, possessing bulky connecting groups, when compared to biphenyl derivatives.

The anti-proliferative and pro-apoptotic properties displayed by these compounds are a proof-of-concept that the removal of CA4’s ethylene does not completely abolish *in vitro* anti-cancer activity. Importantly, the *in vivo* anti-cancer activity of CA4P has been shown to be largely independent of direct cancer cell targeting and instead is linked to its vascular damaging properties [50]. Thus, future characterization of MP5-G9 and MP5-59, as well as future biphenyl analogues, will need to involve animal models to evaluate the vascular damaging properties, bioavailability, and therapeutic potential.

## Supporting information

S1 Fig CA4 and biphenyl analogues reduce growth and proliferation of colorectal cancer spheroids. Saos-2 osteocarcoma cells were grown in ultra-low adherent round-bottomed 96-well plates for 48 hours to establish spheroid formation. Subsequently, spheroids were treated with indicated dose of either CA4 or biphenyl derivative for 72 hours. Spheroid morphology and size was monitored via differential interference contrast microscopy. Scale bar = 500 microns.
(TIF)

S2 Fig CA4 and biphenyl analogues do not induce mitochondrial collapse and apoptotic induction at early time points. (A) Treated MV-4-11 cells were probed with tetramethylrhodamine methyl ester (TMRM). Taxol treated cells served as positive controls. Percentage of TMRM positive cells was quantified using image-based cytometry. Values are expressed as mean ± SD of three independent experiments. (B) Treated MV-4-11 cells were probed with annexin V and propidium iodide (PI). Percentage of annexin V and PI positive cells was quantified using image-based cytometry. 1.0 μM staurosporine (STS) treated cells served as a positive control. Blue bar represents viable cells; orange bar represents PI and annexin V positive cells; green bar represents annexin V positive cells; red bar represents PI positive cells. Values are expressed as mean ± SD from three independent experiments. * *p* < 0.05 vs. DMSO control.
(TIF)

S3 Fig Mitotic arrest following treatment of MV-4-11 cells with CA4 and biphenyl analogues precedes DNA damage. Treated MV-4-11 cells (dose and time are indicated) were fixed overnight with ice cold 80% ethanol and stained with propidium iodide. Cell cycle profiles were generated using image-based cytometry. Area shaded with red lines slanted downwards to the right = G1/G0; area shaded with vertical red-lines = S phase; area shaded with red lines slanted downwards to the left = G2/M; area shades with blue hatches = cells with damaged nuclei.
(TIF)

S4 Fig Small molecule inhibitors of mitotic arrest able to ameliorate mitotic arrest, mitochondrial collapse, and apoptotic induction. E6-1 cells were treated with either CA4, MP5-F9, MP5-G9, or nocodazole (NOC), alone or co-incubated with either reversine (rev) or RO-3306, for 24 hours. (A) Whole cell lysates were electrophoresed, transferred to a PVDF membrane and probed for cyclin B1 and β-actin. Bands were visualized with enhanced chemiluminescence reagent. Western blot images are representative of two independent trials. (B) Treated cells were probed with tetramethylrhodamine methyl ester (TMRM). Percentage of TMRM positive cells was quantified using image-based cytometry. Values are expressed as mean ± SD of at least three independent experiments. * *p* < 0.05; **** *p* < 0.0001. (C) Treated cells were probed with green fluorescent annexin V and propidium iodide (PI), a red fluorescent probe. Percentage of annexin V and PI positive cells was quantified using image-based cytometry. Blue bar represents viable cells; orange bar represents PI and annexin V positive cells; green bar represents annexin V positive cells; red bar represents PI positive cells. Values are expressed as mean ± SD from at least three independent experiments. *** *p* < 0.001; **** *p* < 0.0001.
(TIF)

S5 Fig Chemical structures of CA4 and colchicine biphenyl analogues. Two representative and biologically inactive biphenyl CA4 analogues are depicted. Additionally, a biologically active biaryl colchicine analogue is presented.
(TIF)
