# Supplementary figures and images for: Structurally simplified biphenyl combretastatin A4 derivatives retain in vitro anti-cancer activity dependent on mitotic arrest
Source: PLoS One. 2017 Mar 2;12(3):e0171806. doi: 10.1371/journal.pone.0171806 (PMC5333808; doi:10.1371/journal.pone.0171806)

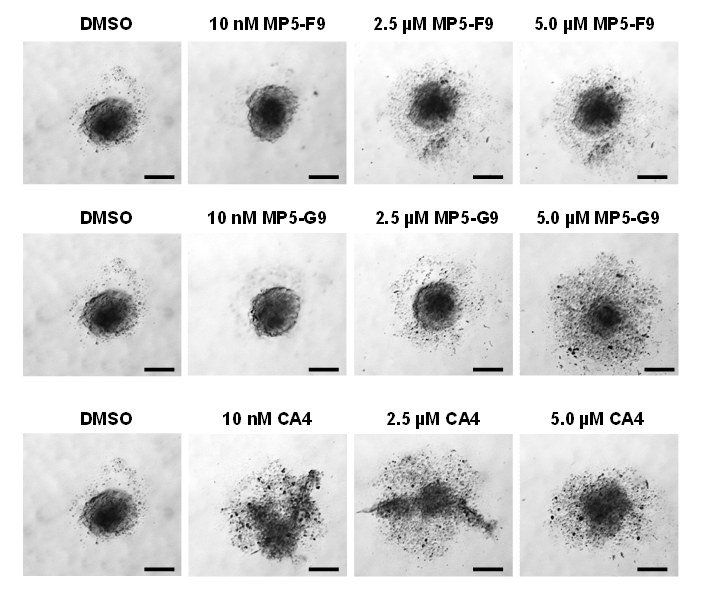

Supplement: S1 Fig — Saos-2 osteocarcoma cells were grown in ultra-low adherent round-bottomed 96-well plates for 48 hours to establish spheroid formation. Subsequently, spheroids were treated with indicated dose of either CA4 or biphenyl derivative for 72 hours. Spheroid morphology and size was monitored via differential interference contrast microscopy. Scale bar = 500 microns. (TIF) [file pone.0171806.s001.tif]

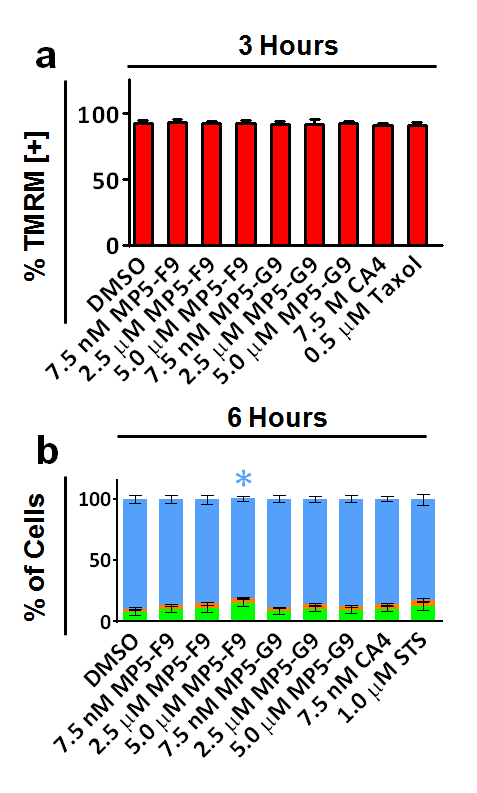

Supplement: S2 Fig — (A) Treated MV-4-11 cells were probed with tetramethylrhodamine methyl ester (TMRM). Taxol treated cells served as positive controls. Percentage of TMRM positive cells was quantified using image-based cytometry. Values are expressed as mean ± SD of three independent experiments. (B) Treated MV-4-11 cells were probed with annexin V and propidium iodide (PI). Percentage of annexin V and PI positive cells was quantified using image-based cytometry. 1.0 μM staurosporine (STS) treated cells served as a positive control. Blue bar represents viable cells; orange bar represents PI and annexin V positive cells; green bar represents annexin V positive cells; red bar represents PI positive cells. Values are expressed as mean ± SD from three independent experiments. * p < 0.05 vs. DMSO control. (TIF) [file pone.0171806.s002.tif]

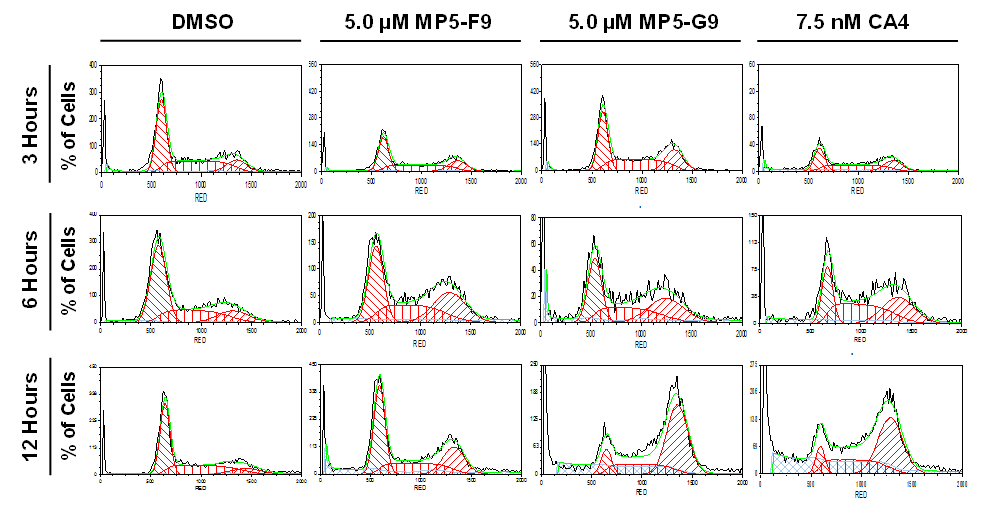

Supplement: S3 Fig — Treated MV-4-11 cells (dose and time are indicated) were fixed overnight with ice cold 80% ethanol and stained with propidium iodide. Cell cycle profiles were generated using image-based cytometry. Area shaded with red lines slanted downwards to the right = G1/G0; area shaded with vertical red-lines = S phase; area shaded with red lines slanted downwards to the left = G2/M; area shades with blue hatches = cells with damaged nuclei. (TIF) [file pone.0171806.s003.tif]

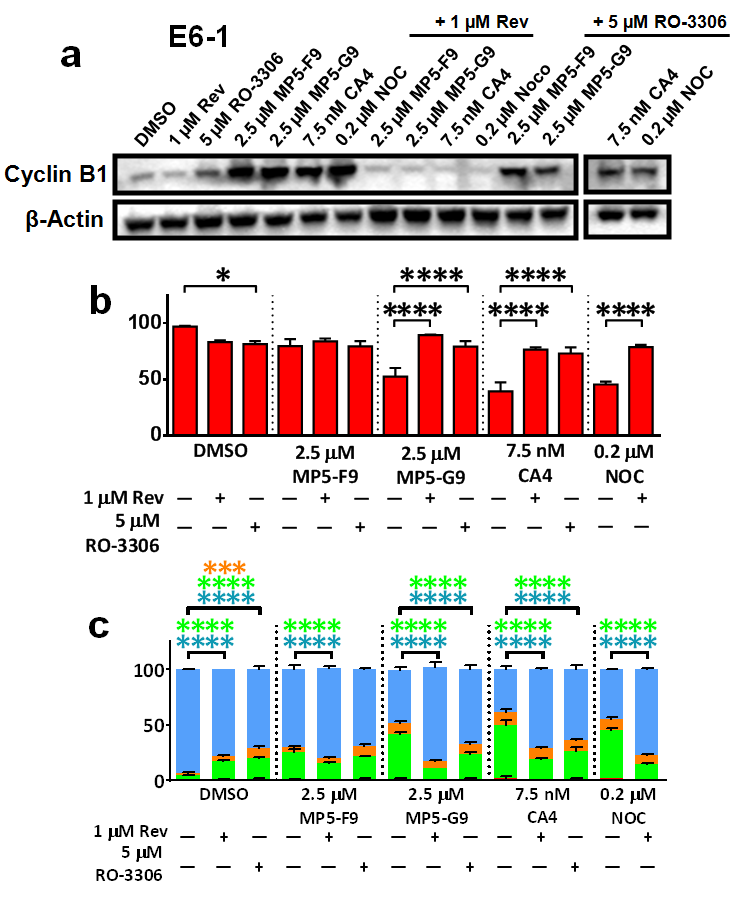

Supplement: S4 Fig — E6-1 cells were treated with either CA4, MP5-F9, MP5-G9, or nocodazole (NOC), alone or co-incubated with either reversine (rev) or RO-3306, for 24 hours. (A) Whole cell lysates were electrophoresed, transferred to a PVDF membrane and probed for cyclin B1 and β-actin. Bands were visualized with enhanced chemiluminescence reagent. Western blot images are representative of two independent trials. (B) Treated cells were probed with tetramethylrhodamine methyl ester (TMRM). Percentage of TMRM positive cells was quantified using image-based cytometry. Values are expressed as mean ± SD of at least three independent experiments. * p < 0.05; **** p < 0.0001. (C) Treated cells were probed with green fluorescent annexin V and propidium iodide (PI), a red fluorescent probe. Percentage of annexin V and PI positive cells was quantified using image-based cytometry. Blue bar represents viable cells; orange bar represents PI and annexin V positive cells; green bar represents annexin V positive cells; red bar represents PI positive cells. Values are expressed as mean ± SD from at least three independent experiments. *** p < 0.001; **** p < 0.0001. (TIF) [file pone.0171806.s004.tif]

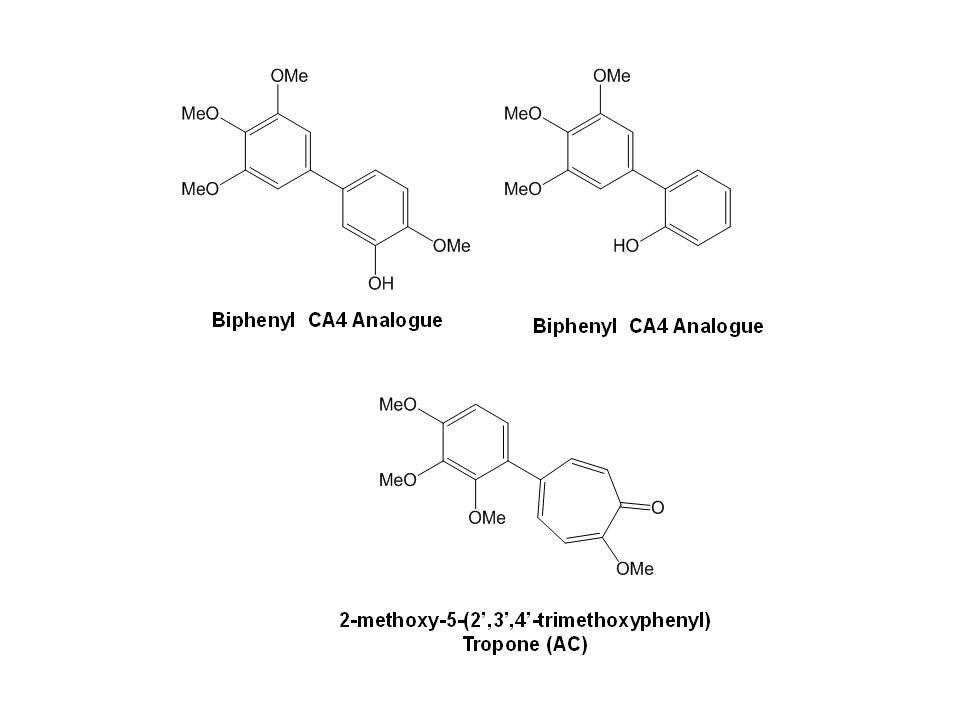

Supplement: S5 Fig — Two representative and biologically inactive biphenyl CA4 analogues are depicted. Additionally, a biologically active biaryl colchicine analogue is presented. (TIF) [file pone.0171806.s005.tif]
